# Challenges in Providing Treatment and Care for Viral Hepatitis among Individuals Co-Infected with HIV in Resource-Limited Settings

**DOI:** 10.1155/2012/948059

**Published:** 2012-03-26

**Authors:** Wirach Maek-a-Nantawat, Anchalee Avihingsanon, Pirapon June Ohata

**Affiliations:** The HIV Netherlands Australia Thailand Research Collaboration, Thai Red Cross AIDS Research Centre, 104 Rajdumri Road, Pathumwan, Bangkok 10330, Thailand

## Abstract

Hepatitis B and C infections are prevalent among HIV-infected individuals with different epidemiologic profiles, modes of transmission, natural histories, and treatments. Southeast Asian countries are classified as “highly prevalent zones,” with a rate of hepatitis B and C coinfection in people living with HIV/AIDS of approximately 3.2–11%. Majority of hepatitis B coinfection is of genotype C. Most of the patients infected with hepatitis C in Thailand have genotype 3 which is significantly related to intravenous drug use whereas, in Vietnam, it is genotype 6. The options for antiretroviral drugs are limited and rely on global funds and research facilities. Only HBV treatment is available for free through the national health scheme. Screening tests for HBV and HCV prior to commencing antiretroviral treatment are low. Insufficient concern on hepatitis-virus-related liver malignancy and long-term hepatic morbidities is noted. Cost-effective HCV treatment can be incorporated into the national health program for those who need it by utilizing data obtained from clinical research studies. For example, patients infected with HCV genotype 2/3 with a certain IL-28B polymorphism can be treated with a shorter course of interferon and ribavirin which can also help reduce costs.

## 1. Introduction

Most countries in Southeast Asia currently have limited resources in providing universal coverage for HIV treatment and care. Since the start of the global AIDS epidemic, Thailand has become the only country within this region with a high prevalence of HIV infection (>1%). In 2009, the HIV prevalence has decreased to 2.3 million because of the HIV prevention campaigns. These HIV prevention campaigns focused on promoting the use of condoms among commercial sex workers and their clients which achieved >90–95% success in preventing HIV transmission. Another campaign known as the prevention of mother-to-child transmission (PMTCT) was also successful. However, in recent years, the rates of new HIV infection have increased among hard-to-reach groups such as the men having sex with men (MSM), injection drug users (IDUs), and adolescents. It has been shown that only 20–30% of sexually active young people used condoms consistently. Furthermore, these HIV-infected high risk groups have sexually transmitted hepatitis infection, especially HCV.

Antiretroviral drugs are provided through the national health schemes and international funding agencies such as the Global Fund and PEPFAR. These antiretroviral therapy (ART) programs provide financial support for ARV protocol development, professional health care training, drug supply chain management, formation of a laboratory network, monitoring and evaluation, and other needs requested by the multisector and various people living with HIV/AIDS (PLWHA) groups. The program has scaled up but mostly lacks local leadership, comprehensive training, and coordination to achieve and sustain the success of the program. Furthermore, patients coinfected with hepatitis are currently being ignored. Screening and treatment for hepatitis coinfection should be included in the national policy to reduce problems in the future.

## 2. Epidemiology of HBV and HCV Coinfection

It has been documented that HBV infection in the general population is high (>8%) in many countries from the Southeast Asia region [1–4]. Reports showing lower infection rates of 3.2–6% from Brunei Darussalam, Indonesia, Philippines, and Thailand [5–9] are based on cases infected through vertical transmission. In tertiary care settings, HBV infection in HIV-infected population was approximately 8.7–11% [10–14]. MSMs and IDUs are at a higher risk of being infected with HBV and HIV. However, the infection rate of HBV in PLWHA is not that much different compared to the general population of which majority are infected perinatally. The most common HBV genotypes are C [15–17] and B [18, 19] followed by A. The most common genotypes found among migrants living in Thailand are C (86%) and B (11.6%) [20]. The prevalences of HBsAg among migrants from Cambodia, Laos, and Myanmar living in Thailand were 10.8%, 6.9%, and 9.7%, respectively [20]. In the last 10 years, chronic perinatal infection has decreased when the national program expanded its immunization protocol to include HBV vaccination in all children. Even though the HBV subtypes of the surface antigen [15, 18, 21] reported are *adr* and *adw*, this is not clinically significant because the HBV vaccine of either *adw* or *ayw* subtype can yield anti-*a* which is protective against crossinfection with other HBsAg subtypes as well [22].

In contrast to HBV, HCV co-infection is moderately high in PLWHA, especially among IDUs and MSMs. If HIV prevalence in IDUs is high, eventually HCV co-infection will become a major problem as currently seen in Thailand and Vietnam [23]. Among PLWHA, it has been estimated that approximately 5–40% have contacted HIV from injecting drugs. In Thailand and Vietnam, at least 50% of IDUs are living with HIV/AIDS, and about 90–95% have also contracted HCV. The estimated prevalence of HCV/HIV co-infection is 7.2–10.1% [10, 11, 14, 24, 25]. Unlike Thailand and Vietnam, in the Philippines, 83–89% of IDUs are infected with HCV whereas only 0.34% are infected with HIV. Hence, the prevalence of HCV/HIV co-infection is low [26]. Factors such as males [10, 27] and IDUs are at higher risk of becoming infected with HCV and HIV. Among the general population, the most common genotypes of HCV in Thailand, Vietnam, and Indonesia are 3a (70%), 6a (32.5%), and 1b (47.3%), respectively [25, 28, 29]. However, in HIV-infected individuals, genotype 1 is increasingly found [27]. The prevalence of HIV-HBV-HCV triple infection is rare (0.4%) but can be found among IDUs and MSMs [11]. The predominant HCV genotypes detected in migrants from Cambodia, Laos, and Myanmar living in Thailand were 1a, 1b, 3, and 6, respectively. However, this data may not accurately reflect the real HCV genotypes among these groups of people because few samples were collected from that study [30].

## 3. Approach in Diagnosing HBV or HCV among HIV-Infected Patients

Many individuals infected with HBV at birth or during early childhood and subsequently infected with HIV have asymptomatic HBV chronic infection with or without aminotransaminase elevation. Patients who develop acute hepatitis B during their adulthood will have abrupt and progressive jaundice with GI symptoms such as nausea, abdominal pain, flatulence, and bloated abdomen. The general symptoms include fatigue, dizziness, weight loss, or anorexia. The liver is usually not enlarged, and the cutaneous stigmata of chronic liver disease is not detected unless the disease has progressed to decompensated cirrhosis. Cirrhosis is more common in patients with lower levels of ALT and CD4 compared to those monoinfected with HBV [31, 32]. HIV-HBV co-infected men are much more likely to die of liver-related causes compared to monoinfected HBV patients [33]. The risk of HCC is somewhat increased in HIV-infected individuals with low CD4 counts [34]. Patients with genotype C have exhibited earlier progression of cirrhosis and HCC than those with genotype B [17].

Similarly, patients co-infected with HCV and HIV have asymptomatic acute HCV infection. It is also possible that many IDUs co-infected with HCV and HIV may not have reported their symptoms, and this may not necessary reflect an accurate account of HCV infection among these groups of people. Usually, in non-IDUs, acute hepatitis C is detected in PLWHAs currently on treatment and diagnosed with sexually transmitted infections (e.g., syphilis, gonorrhea) because of elevated enzyme levels. HIV positive individuals with acute HCV infection can develop chronic HCV infection. In contrast to HBV, in HCV co-infected patients, disease progression to liver cirrhosis and hepatocellular carcinoma (HCC) occurs very quickly and may exist prior to HCV treatment. As a result of this, physicians need to closely monitor these patients, even those that have sustained viral response to HCV treatment. Routine HBV and HCV screening are not routinely performed at tertiary care setting. Many HIV patients with undetectable HIV RNA and elevated liver enzymes are screened for hepatitis and eventually found to be co-infected with HBV [14, 35].

Currently, the national guidelines for antiretroviral therapy in HIV-infected adults and adolescents in most countries recommend HIV-infected persons to be screened for HBV before initiating ART. The reason for this is because this will help guide physicians in designing the patient's HAART regimen which should contain at least 2 antiretroviral drugs with activity against both HIV and HBV, that is, tenofovir plus lamivudine or tenofovir plus emtricitabine. The viral hepatitis serology is widely available but not routinely used to screen those patients most at risk such as IDUs and MSMs. Tests for HBs antigen are recommended to all HIV-infected patients, but recently, in actual practice, 55–69% of HIV-infected adults were tested for hepatitis [12, 13, 36]. Asymptomatic chronic infected cases are not unmasked and may continue to transmit the viruses through contaminated blood and genital secretions. If HIV-infected people are aware of the effects of HBV and the accessibility of HBV treatment, then the rate of HBV screening prior to ART may improve. The results of HBV serologic profiles are interpreted as mono-HBV infection (see Table 1). However, isolated positive core antibodies are more frequently (20–30%) found in co-infected patients [37] compared to those monoinfected with HBV, especially in advanced immunocompromised [38], HCV co-infected cases [39–41], or IDUs. The clinical significance of having a positive anti-HBc antibody is not well understood, but there is more evidence indicating that people with this serologic finding has an occult infection with frequent hepatic flares [42] and potential of transmitting the infection [43–46]. In certain cases, some may have undetectable HBV DNA with isolated core antibody [47, 48].

Since HBV/HIV co-infection is common, it is highly recommended that in every HIV-infected individuals, serologic screening tests for hepatitis B should include HBs Ag, anti-HBs, and anti-HBc antibody. If all 3 serologic tests are negative, then it is highly recommended that the patients get a hepatitis B vaccine to prevent infection. If an isolated core antibody is detected, then a confirmatory HBV-DNA or complete liver function workup is needed to help guide the patients' long-term care management. It is not conclusive whether HIV-infected individuals with isolated core antibody should get a Hepatitis B vaccine. The vaccination may have a primary or an amnestic response [37]. If HBs antigen is positive, then it is important to assess whether the patient also has HBe Ag, anti-HBe antibody, HBV-DNA and liver enzymes to rule out viral replication, liver complications and whether treatment is needed.

Majority of patients presenting acute HBV infection will have elevated levels of liver enzymes (ALT > AST with >10 times ULN). However, as for those currently infected with HBV or have tested positive for HBs antigen, it is difficult to differentiate whether they are HBV carriers or have chronic infection with low or nonreplicative phase. In healthy carriers, HBV-DNA may not be detectable because of transient viremia and therefore would require retesting. HBV-DNA is somewhat useful but is too expensive for some countries with limited resources. Certain places may not have access to machines to detect HBV-DNA, and some patients may not be able to afford such diagnostic tests. In order to detect and differentiate chronic active HBV patients from positive serostatus for HBe Ag, physicians would need the patients' medical history, risk behaviors or predisposing factors, and physical findings such as stigmata of chronic liver.

Liver enzymes can be used as a surrogate marker for detecting hepatic necroinflammation, but its elevation may also indicate a hepatic flare from any causes including the virus itself. Serum aminotransferase levels are less reliable in determining whether the patient would need therapy or not. Serum aminotransferase levels can be lower in patients co-infected with HIV and HBV or within normal range in some patients with significant hepatic fibrosis. Even though liver pathology can specifically detect fibrosis and necroinflammation by using a scoring system; however, its procedure is invasive, time-consuming and may not be sensitive if there is bias in the way the samples are collected or assessed. Assessing the extent of the underlying liver damage is important because it will affect the prognosis of the infection as well as the choices for treatment. Another noninvasive test known as the hepatic elastography (Fibroscan) can be used to measure the liver's stiffness or evaluate hepatic fibrosis. The results from the Fibroscan can guide treatment and care but may not be possible in resource-limited settings. Some of the limitations of the fibroscan are its inability to accurately predict the degree of injury seen on a liver biopsy or subsequent clinical events. Close monitoring is necessary to detect early cases.

In regards to HCV co-infection, the national guidelines recommend to screen for anti-HCV antibody before initiating ART in HIV-infected adults exhibiting symptoms or those with risk factors such as intravenous drug use. The treatment cost for HCV is extremely expensive and is not covered by the national health schemes. Patients with HCV who need treatment must pay for their own treatment. Aside from that, the national health schemes do not provide free diagnostic tests for HCV genotype and HCVRNA load. At the present, in majority of the countries, there is insufficient epidemiological data on HIV/HCV co-infection. Hence this may be one of the reasons why the national health schemes will not offer free HCV testing in HIV-infected individuals. According to the physicians who have done anti-HCV tests in their HIV-infected patients, there is a high prevalence of HCV co-infection. This result indicates that an appointed committee should include HCV tests in the national guidelines for HIV-infected patients.

It is possible to use tests to detect for anti-HCV antibody to screen those groups at risk for acquiring the infection. However, it is important to note that anti-HCV seroconversion occurs much slower in HIV-infected patients, and it is still possible to have anti-HCV negative results despite ongoing viral replication for a year [49]. Currently, HCV antibody tests cost around 6–9 USD. In a resource-limited setting, this cost is affordable yet it is not included in the national health care program. This test can be used to screen and diagnose HCV in HIV-infected patients even though it may not be perfect. It can be used in clinical settings or as requested. In most of the cases, there are no or very little clinical symptoms as seen in people with acute HCV infection. Therefore, acute HCV infection is defined as having detectable HCV-RNA in the first 6 months after infection. Transaminase levels can also be used to accurately detect acute HCV infection. Elevated levels of alanine transaminase (ALT) are more sensitive in detecting acute HCV when compared to anti-HCV antibody tests. Tests to detect HCV-RNA are used to determine the virus's replicative state. Hence results from HCV-RNA tests can detect early infection better than the antibody tests and are usually used to exclude false positive results obtained from the serologic tests when the patients have disclosed not having any risk behaviors. Sometimes it is also used to determine whether the result from the serology test is a false positive or not. Past resolved HCV infection may yield false positive results in the serology test. HIV positive individuals who need to start antiviral treatment should be screened for HCV by using the HCV-RNA tests and monitored regularly [50]. Chronic hepatitis C infection is defined as having ongoing viral replication for more than 6 months. Without the patient's past hepatitis C test results, it is difficult to determine the HCV status of the patient based only on the patient's history, current physical and laboratory examinations. It is very difficult to distinguish between acute and chronic infection because flares during chronic hepatitis C may mimic acute infection.

**Figure 1:**
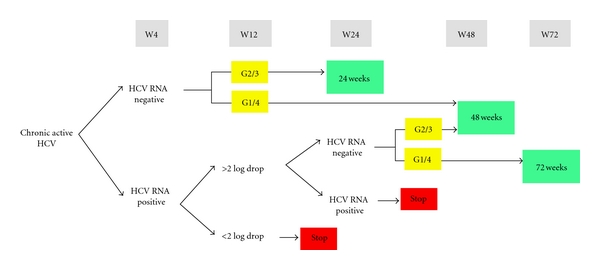
Treatment scheme for HCV coinfection guided by genotype and HCV-RNA load assessment at baseline, wks 4, 12, and 24 [48, 72].*Patients having baseline HCV-RNA load <400,000 IU/mL with minimal fibrosis.

Genotyping should be done in every case who will need HCV treatment because this will help guide the physician in determining the length of treatment, predict treatment response and prognosis of HCV (see Figure 1). However, if genotyping tests are not available, then physicians from resource-limited setting can use regional epidemiological data to determine the subtype of HCV. As for those patients not on HCV treatment due to no indication, treatment intolerability or failure and drug availability, it is important to continue to monitor and assess the progression of the disease. Liver enzymes should regularly be checked every 3 months and repeated if there are significant elevations. The degree of histologic injury is a better predictor of subsequent clinical events than is the degree of elevated serum aminotransferase levels, genotype, or viral load. The result of the Fibroscan can support the physician's decision to start or defer treatment with a higher level of confidence. CD4 cell count appears to be a good predictor for spontaneous clearance [51]. Antiretroviral treatment may help to control HCV if HCV treatment is not provided. Thrombocytopenia and reversed albumin/globulin ratio can be detected in cases with progressive liver cirrhosis in patients with hepatitis C. Also, serological markers correlated with stages of liver fibrosis can be used with indices obtained from routine blood tests to determine the function of the liver (e.g., APRI (aspartate aminotransferase [AST]-to-platelet ratio index) and Fib-4 (age, AST, platelets, and ALT level)). Alpha feto-protein (AFP), a tumor marker for hepatocarcinoma, can help interpret liver images whereas pathology can confirm the stage of the disease. Screening for hepatocarcinoma should include ultrasound of the liver and serum AFP should be performed every 6 months for all chronic hepatitis B and C patients with cirrhosis. However, according to the systematic reviews, AFP is not considered sensitive (73.5%) [52, 53] and has no existing correlations in hepatocarcinoma [54]. This serves as a warning sign that more needs to be done to prevent the transmission of the virus as well as increase the community's awareness of comorbidity among hepatitis co-infected patients.

In conclusion, routine screening using serologic tests for hepatitis B and C is beneficial for the patient to determine when to start treatment or those who cannot afford such care to closely monitor the disease progression and complications. The use of stavudine, didanosine, and nevirapine, which are unfriendly to the liver, should be used with caution because it can lead to liver toxicities. For public health concerns, this will also help reduce the risk of transmission if treatment is provided and reduce risk behaviors. As for those at risk of acquiring hepatitis such as immunocompromised patients, physicians can recommend HBV vaccinations. It is highly recommended that in resource-limited settings where there is a high prevalence of hepatitis, HBV and HCV should be screened in HIV-infected patients prior to ART.

## 4. Management of Coinfected Patients

Recently, the international HIV treatment guidelines 2011 recommend that antiretroviral therapy should be started in all HBV/HIV co-infected adolescents and adults who require treatment for chronic active hepatitis B irrespective of their CD4 cell counts. According to these new guidelines, all HIV-infected individuals with CD4 ≤ 350 cells/*μ*L are required to start antiretroviral therapy regardless of symptoms. However, in the middle of 2011, there is evidence that this is not implemented throughout the region. Therefore, it is important to assess the treatment rates in cases with CD4 of 200–350 cell/*μ*L to ensure treatment coverage and care among these people in order to reduce opportunistic infections in severely immunocompromised patients. Some nucleoside/nucleotide analogs for HIV treatment are effective to both HIV and HBV and therefore can be used to treat HIV/HBV co-infected patients. Furthermore, the results from the study on Tenofovir in HBV Coinfection (TICO) which was conducted in Thailand showed that a combination of tenofovir and emtricitabine or lamivudine was better than using only tenofovir [55, 56]. There was an increased loss of HBeAg when a longer follow-up period was implemented to assess HBV treatment outcome. Hepatic flares were observed in 19–25% of the patients without any severe complications. Interestingly, long-term use of tenofovir in HIV/HBV co-infected patients may prevent disease progression to end-stage liver disease in the Thai population, by slowing or reversing liver fibrosis. Currently, the Thai national guideline recommends using tenofovir with either lamivudine or emtricitabine for any HBV co-infected individuals regardless of their baseline CD4+ T-cell count. Since HBV treatment is cheap (approximately 55 USD/mo. for tenofovir plus lamivudine and 70 USD/mo. for tenofovir plus emtricitabine), this is covered by the national health scheme. However, the cost for monitoring HBV-DNA is not included in the national AIDS program, and the patients have to pay for this by themselves. Other nucleoside analogs such as adefovir, telbivudine, and entecavir are also not included because the government has decided that these drugs are not essential for the mass treatment of HBV. Moreover, it is still unclear which HBV drugs should be used for the preferred regimen in pregnant women, and infants born to HBV co-infected mothers. 

The situation is even worse for those co-infected with HIV and HCV. The current national treatment guideline recommends pegylated interferon *α*2a or 2b plus ribavirin (Figure 1). Pegylated interferon and ribavirin are not on WHO and Asian national essential medicines lists for all HCV patients and hence are not freely provided through the national health schemes. As for other low and middle-income countries, many patients cannot afford pegylated interferon and ribavirin due to its costs. Currently, patented pegylated interferon from Roche (Pegasys) and Merck (PegIntron) is packaged and sold with generic ribavirin. Because of these patents, hepatitis C treatment remains to be expensive. For a 48-week treatment course, it costs approximately $26,000–30,000 USD. Thus, most health care systems cannot provide treatment for HCV and will refuse to offer treatment to majority of patients with HCV. This price does not include other related investigations such as HCV genotyping and HCVRNA as well as treatment for unexpected complications. HCV RNA should be assessed before commencing treatment and used to assess the efficacy of the treatment regimen. The most common adverse events are neuropsychiatric symptoms and marrow toxicity which can add to the cost of treatment and contribute to premature treatment termination. Therefore, in order to minimize adverse effects, antiretroviral therapy needs to be adjusted. Concomitant use of zidovudine is contraindicated due to its effect on the bone marrow; bone marrow suppression is worsened by the use of zidovudine. The use of didanosine is also not recommended during HCV therapy due to increased risks of hepatic decompensation. Problems are further exacerbated if the patient is co-infected with HCV and HIV and receiving treatment concomitantly. It is not possible for all HCV/HIV co-infected patients to get HCV treatment because it is very expensive and has several intolerable side effects. Patients on ART also suffer from pill burdens and side effects of antiretroviral drugs. 

As a result of this, images of the liver by using ultrasound, levels of serum aminotransaminase, and *α*-fetoprotein are regularly monitored in HCV co-infected patients (see Table 2 for summary). Liver biopsy is rarely done to avoid complications and risks for contamination, especially in HIV-infected treatment-naïve patients. Most Thai patients with HCV infection have genotype 3, the type which responds well to treatment, allowing physicians to reduce the treatment duration to 12–16 weeks in those achieving rapid virological response (RVR); this is based on the findings that 82% of patients were successfully treated for HCV which is comparable to a 24-week treatment course [57] and is still also cost-effective if retreating the cases with relapses for 24 weeks [58]. During treatment, RVR measured at week 4 is a strong predictor of sustained virological response (SVR) [59]. Because of this, short-course treatment for 24 weeks is recommended to the patients infected with genotype 2/3, and a shorter treatment course (12–16 weeks) may be an option for patients unable to tolerate treatment with close RVR monitoring. The response-guided therapy aims to optimize treatment outcomes without compromising SVR rates [60]. 

IL28B gene polymorphisms modulate early virological response to peginterferon/ribavirin treatment and is associated with SVR in patients infected with genotype 2/3 HCV who did not achieve RVR [61]. The favorable CC genotype, as compared to either the CT or TT genotypes, has been associated with a 3-fold increase in the rate for spontaneous clearance of HCV [62] and 2-3 folds higher rate of SVR in HCV genotype 1 chronically infected individuals treated with combination pegylated interferon/ribavirin therapy [63]. In contrast to HCV genotype 1 patients, despite the faster initial viral response in the patients carrying C/C, SVR rates of mono-HCV genotype 3 infection were not different compared to the patients carrying T-allele [64, 65]. Quantitative evaluation of interferon-*γ*-inducible protein-10 (IP-10) may add on the predictive value of IL28B polymorphisms for HCV treatment responses [66]. The clinical outcomes of an earlier viral decline and a shorter course treatment in CC patients infected with HIV/HCV genotype 2/3 are warranted. Nonetheless, access to treatment in Thailand is still hindered by the costs of the medications. Furthermore, drug toxicities may contribute to incomplete treatment for HCV among HIV-infected individuals. In order to sustain the effectiveness of HCV treatment, evidence base information on epidemiology and IL28B polymorphism in specific population can be used to minimize the duration of treatment but may compromise the cost instead. Therefore, policy makers need to strongly reconsider integrating optimized treatment regimen for HCV co-infection into the national program for the future. 

## 5. Prevention Programs

After successful integration of the national expanded program on immunization (EPI) on HBV immunization, coverage of the vaccinations has increased in most countries up to 80% as seen by the results from many studies on incidence reduction of HBV and hepatocellular carcinoma in the young age group [68–72]. Due to the high prevalence of HBV infection in the region, HBV serology screening prior to vaccination in high risk groups is not necessary, for example, MSMs, IDUs, and health care workers (HCWs). Many adults, including health care workers (HCWs), cannot reimburse for HBV vaccinations. Currently, the guideline recommendation for HBV treatment and care for HCWs is not well defined. If people are aware of the complications and prognosis of chronic hepatitis, this may encourage people to have HBV serology screening and HBV vaccinations in adults older than 30 years old. For postexposure prophylaxis, hepatitis B immunoglobulin (HBIG) is required in cases that have been exposed to blood from HBV-infected individuals and are not immune to HBV according to the postexposure HBV screening test. Occupational risks can be prevented if high risk groups such as HCWs and health-related students have been immunized. This preventive policy targeting professional health care workers (i.e., hospitals and clinics) at risk of acquiring HBV infection should be integrated into the national health care system. Currently, HBV immunization is recommended to all HIV-infected patients who are susceptible and have achieved immunological success after antiretroviral therapy. However, it is important to conduct a serologic test for HBV to confirm the person's immune status before vaccination because low CD4 levels or the use of NNRTI may affect the response to the vaccine [67]. It has been shown that in HIV-infected individuals, the immune response of generating HBs antibodies was 71.4% which was much lower compared to healthy HIV seronegative individuals. However, no adverse event has been detected. 

Since HCV vaccines are not available for the prevention of the disease, hence it is essential for all high risk groups, including MSMs and IDUs, to continuously receive updated information on HCV transmission and outcomes to reduce their behavior risk of blood-to-blood contamination. Lack of free access to clean injecting equipment for IDUs may not be a critical issue because syringes and needles are available cheaply in drug stores. However, due to social stigmatization, discrimination, and illegal issues, IDUs are afraid to access clean needles and syringes resulting in higher rates of HIV and HCV infection. Even though the Needle and Syringe Program, a harm reduction effort, is successful in Australia in preventing HCV and HIV infection among IDUs, but such a program is difficult to launch in resource-limited setting with a conservative society. Hence, continuing education and health promotion are required to provide correct information to the community to change their perception as a preventive strategy. HIV/HCV co-infection among IDUs is a public health emergency that is currently being ignored by the policy makers. The need for proper policy and advocacy are needed and should be presented through health promotion campaigns in collaboration with working groups and peer educators to successfully implement and launch harm reduction programs properly. Community advocates and appropriate waste disposal need to be worked out before rolling out the harm reduction programs. Regular HCV screening for high risk groups, especially HCWs and patients with chronic renal failure on hemodialysis, is necessary. Voluntarily unpaid donor blood must be routinely screened for HBV and HCV serology at every blood bank unit throughout the region as currently being performed by the Red Cross blood banks.

## 6. Perspectives on Hepatitis B and C Coinfection among HIV-Infected Patients for Testing and Treatment

Since HBV co-infection is more chronic, therefore the national guidelines have recommended sufficient and early screening to initiate proper treatment and care. Despite this, there are still problems for those patients who have developed resistance and have limited selection of drugs to choose from and/or intolerability. It will be a continued struggle to provide alternative treatment and other drug choices for these patients. Hepatitis B vaccination should be implemented at all levels of the population, especially high risk groups and health care workers; HBV vaccination is an urgent and necessary action that should be in place in order to reduce HBV infection. The strategic plan must cover adults older than 30 years old who may become infected and transmit the virus to others via the sexual route. Hepatitis B is preventable and immunization is better than acquiring the virus. The cost of the immunization program is incomparable to the people's quality of life. Recently 2 new protease inhibitors, boceprevir and telaprevir, were approved by the US FDA in May 2011 and by the European Medicine Agency (EMA) in August and September 2011, respectively, for HCV treatment. The drugs can increase the efficacy and RVR when used as a triple drug therapy (with pegylated interferon and ribavirin) in HCV patients with genotype 1 [73–77], but the cost is 2-3 times higher than the standard treatment. For these new drugs, the US FDA is concerned about the adverse events such as suicidal tendencies and lack of efficacy in certain groups of people; boceprevir has been shown to cause rash and gout whereas telaprevir has been associated with TB. Both boceprevir and telaprevir can cause anemia in HCV patients. However, it should be noted that anemia is a common laboratory abnormality among patients infected with both HIV and HCV due to treatment; physicians will need to reduce the dose of their HCV medications in patients with anemia [78]. Aside from additional drug toxicities, evidence-based information from monoinfected HCV clinical trials on shorter triple drug treatment, pharmacokinetics guided optimized dose of new drugs, and potential drug-drug interactions warrant further investigations in Asian population living with HIV/AIDS. HCV Direct-Acting Antivirals (DAAs), new polymerase and protease inhibitors that are under clinical investigations with or without interferon, provide HCV patients with more treatment options. Quad therapy (2 different protease inhibitors plus pegylated interferon and ribavirin) is another option that will become available in the future regardless of the cost of the drugs. To improve tolerability and treatment coverage, the interferon-free DAA-based combination therapy may be an alternative choice for some people [79]. 

HCV infection is a curable disease and the international clinical guidelines already have provided recommendations for screening, diagnostics, and treatment for HCV/HIV co-infected patients. Yet majority of the patients, especially IDUs co-infected with HIV and HCV, still have problems in getting the proper treatment and care. The social stigma around drug use pervades many aspects of the society, creating huge barriers that IDUs face when seeking health care. The barriers in the health care setting, including prejudice and stigmatization, are worse than HIV monoinfection because the medical service providers and policy makers have insufficient experience in dealing with co-infected patients resulting in limited care and financial support. Physicians may refuse treatment to IDUs because of their perceptions that these patients have poor adherence to treatment. In fact, treatment adherence among IDUs substantially increased when they have access to health and social services with harm reduction support and mental health care. A challenge can be met through educating medical staff and providing support for patients. Access to treatment and healthcare should be abrogated by national policymakers when it comes to treating HCV in HIV co-infected patients. The main economic benefit to treating people with HCV is that it will lower the cost and amount of medical care needed for HCV in the long term, including treatment for severe liver disease and HCV-related liver malignancy. Moreover, it is absolutely impossible to put a price on the patient's quality of life as it is priceless and invaluable. Successful treatment can also prevent new HCV infections. 

## Figures and Tables

**Table 1:** Findings and interpretations of HBV serologic markers.

HBs Ag	Anti-HBs	Anti-HBc	Hbe Ag	Interpretation
+	−	−	+	Chronic replicative phase or acute infection
+	−	+	−	Chronic nonreplicative/carrier (DNA neg) Flare up (DNA positive)
+	−	−	−	Precore mutants
−	−	+	−	Isolated core antibody Recovery from acute infection Occult infection (DNA positive)
−	+	−	−	Previously immunized with HBV vaccine

**Table 2:** Summary of diagnostic criteria and treatment currently in use as well as its perspectives in management.

Stage of disease	Diagnostic criteria	Current treatment practices	Perspectives in management
Chronic hepatitis B coinfection	(i) HBsAg+ >6 mos (ii) Serum HBV DNA >2,000 IU/mL (10^4^ copies/mL) (iii) Persistent or intermittent ALT/AST elevation (iv) Liver biopsy (done in some) showing chronic hepatitis with moderate or severe necroinflammation	Tenofovir plus lamivudine or tenofovir plus emtricitabine	(i) HBV-DNA assessment for treatment outcome (ii) Add adefovir or entecavir if no virologic suppression or suspected resistance (iii) Close monitoring of cirrhosis and hepatocellular carcinoma (iv) Hepatitis B vaccination for susceptible partner

Inactive HBsAg carrier state among PLWHA	(i) HBsAg+ >6 mos (ii) HBeAg−, anti-HBe+ (iii) Serum HBV DNA <2,000 IU/mL (10^4^ copies/mL) (iv) Persistently normal ALT/AST levels (v) Liver biopsy (unfortunately not done in clinical practice as recommended) confirms absence of significant hepatitis	Due to limited options of antiretroviral regimen, lamivudine is used as part of HAART in majority of cases that need HIV treatment	(i) Misleading term/new term “chronic low replicative hepatitis B” can be used (ii) Lamivudine/emtricitabine preserved for combination treatment for HBV infection if indicated (iii) Need to closely F/U: LFT. *α*-FP and ultrasound regularly at least q 6–12 mos for cirrhotic patients (iv) Hepatitis B vaccination for susceptible partner

Occult hepatitis B coinfection or isolated core antibody	(i) Presence of anti-HBc +/− anti-HBs (ii) HBsAg− (iii) Undetectable serum HBV DNA (very low levels may be detected by sensitive PCR assays) or serum HBV DNA <2,000 IU/mL (10^4^ copies/mL) (iv) Normal ALT levels	Due to limited options of antiretroviral regimen, lamivudine is used as part of HAART in majority of cases that need HIV treatment	(i) Lamivudine/emtricitabine preserved for combination treatment for future HBV infection if occult infection suspected (ii) LFT q 6 mos if ALT/AST elevated, further assessment for HBe Ag and HBV-DNA (iii) Hepatitis B vaccination for susceptible partner (iv) It is not clear whether Hepatitis B revaccination is needed or not

Chronic hepatitis C coinfection	(i) Anti-HCV+ and HCV-RNA+ (ii) Normal ALT levels or ALT elevation (iii) Liver biopsy showing fibrosis (or Fibroscan >7.5 kPa)	No treatment in most cases For those who can afford treatment: PegIFN*α*2a or 2b plus ribavirin 800 mg/D, duration of treatment guided by genotype: 3,6 for 24 wks; 1 for 48 wks	(i) Selected cases with good prognostic factors can access treatment comprising PegIFN plus RBV (ii) Genotyping and HCV-RNA for assessing EVR, RVR, and SVR (iii) Lower dose of PegIFN (iv) Shorter treatment duration (v) Need F/U: LFT, *α*-FP and ultrasound q 6–12 mos for cirrhotic patients (vi) Close monitoring of cirrhosis and HCC (vii) Harm reduction to reduce transmission
